# Urinary tract infections during pregnancy – clinical picture and therapeutic approach in the Infectious Diseases Hospital of Iaşi (2009-2012)

**DOI:** 10.1186/1471-2334-13-S1-O9

**Published:** 2013-12-16

**Authors:** Mihaela Cătălina Luca, Andrei Vâță, Olivia Dorneanu, Jugănariu Gabriela, Carmen Dorobăț

**Affiliations:** 1 “Gr.T.Popa” University of Medicine and Pharmacy, Iaşi, Romania; 2 Infectious Diseases Hospital Iaşi, Romania

## Background

Urinary tract infections in pregnancy represent one of the significant pathologies that can influence both the health of the mother and of the child, often giving rise to a polymorphic clinical picture and posing therapeutic difficulties, due to antibiotic resistance and drug toxicity.

## Methods

We performed a retrospective study on 60 pregnant patients, diagnosed with urinary infections in the Clinic Hospital of Infectious Diseases of Iaşi, between January 2009 and December 2012.

## Results

The annual number of the admissions was relatively constant, except for the year 2010 with 25 cases; the infections were more frequent in 26-40 years old pregnant women (63.3%), during the first (38.3%) and the second trimester of pregnancy (48.3%) and in primiparous women (61.7%). Fever was present in 71.7% of the patients, 51.7% having also digestive symptomatology. The inflammatory syndrome was illustrated at admission through leukocytosis (in 66.7% cases), high values of ESR (88.9% cases), and of fibrinogen (31% of cases). Pyuria was present in 75% of cases, and in 25.7% were associated albuminuria. 65% cases were considered lower urinary tract infections. The etiology was established in 73.3% cases, being dominated by *E coli* (88.3%); also implicated were: *Klebsiella* spp. (6.7%), *Streptococcus* spp. (1.7%), *Candida* 3.3%. The etiologic treatment was based on beta-lactams, especially in association with beta-lactamase inhibitors: amoxicillin-clavulanate – 43.3%, ampicillin-sulbactam – 3.3%, second generation cephalosporins – 16.7% or third generation cephalosporins (18.3%) and carbapenems – 10%. The evolution was favorable in all the cases, with no immediate negative effects on the baby.

## Conclusion

The urinary tract infections were more frequent during the first months of pregnancy, in primiparous women older than 26 years old, the etiology being dominated by *E coli* with a decreased antibiotic susceptibility in the last years.

